# Membrane of *Candida albicans* as a target of berberine

**DOI:** 10.1186/s12906-017-1773-5

**Published:** 2017-05-17

**Authors:** Nataša Zorić, Ivan Kosalec, Siniša Tomić, Ivan Bobnjarić, Mario Jug, Toni Vlainić, Josipa Vlainić

**Affiliations:** 1 Agency for Medicinal Products and Medical Devices of Croatia, Ksaverska cesta 4, 10000 Zagreb, HR Croatia; 2 University of Zagreb, Faculty of Pharmacy and Biochemistry, Zagreb, Croatia; 3 Department of Molecular Medicine, Ruđer Bošković Institute, POB 180, 10000 Zagreb, Croatia

**Keywords:** Berberine, *Candida albicans*, Antifungal, Membrane

## Abstract

**Background:**

We investigated the mechanisms of anti-*Candida* action of isoquinoline alkaloid berberine, active constituent of medically important plants of Barberry species.

**Methods:**

The effects on membrane, morphological transition, synthesis of ergosterol and the consequent changes in membrane permeability have been studied. Polarization and lipid peroxidation level of the membrane following berberine treatment have been addressed.

**Results:**

Minimal inhibitory concentration (MIC) of berberine against *C. albicans* was 17.75 μg/mL. Cytotoxic effect of berberine was concentration dependent, and in sub-MIC concentrations inhibit morphological transition of *C. albicans* cells to its filamentous form. Results showed that berberine affects synthesis of membrane ergosterol dose-dependently and induces increased membrane permeability causing loss of intracellular material to the outer space (DNA/protein leakage). Berberine also caused membrane depolarization and lipid peroxidation of membrane constituents indicating its direct effect on the membrane. Moreover, ROS levels were also increased following berberine treatment indicating further the possibility of membrane damage.

**Conclusion:**

Based on the obtained results it seems that berberine achieves its anti-*Candida* activity by affecting the cell membrane.

## Background

Opportunistic infections in immunocomprised hosts and growing resistance to existing therapeutics have triggered the need for development of new antimicrobial drugs [1].

Isoquinoline alkaloid berberine is present in root, rhizome and stem bark of medically important plants of Barberry species. It has been traditionally used for many years in Ayurvedic and Chinese medicine as antimicrobial agent [2]. Published studies have reported its antibacterial activity against staphylococcal, streptococcal and enterococcal species, including MDR strains of *Mycobacterium tuberculosis* and MRSA. In vitro studies showed that berberine has activity against clinical isolates of MRSA, with MICs ranging from 32 to 128 μg/mL [3]. Berberine was also effective in protecting mice infected with *Salmonella typhimurium*: 50% of mice that were not treated with berberine died by the end of the eight day after infection [4]. In combination studies, synergism of berberine was demonstrated with amphotericin [5], fluconazole [6] and miconazole [7] what offers a new approach in the treatment of opportunistic infections resistant to antibiotics. It was reported that berberine loweres MICs of ampicillin and oxacillin against MRSA. Concentrations of 1–50 μg/mL berberine decreased levels of MRSA adhesion and intracellular adhesion compared with the control group [3]. There is also evidence suggesting that bacteria do not develop resistance to berberine since MIC of berberine within same bacterial cultures (*E. coli*, *S. aureus*, *Bacillus subtilis*, *Proteus vulgaris*, *S. typhimurium* and *P. aeruginosa*) did not increase over 200 generations [8]. Efficacy of berberine against *Candida* species [9, 10] has encouraged us to investigate further its mechanism of action against *C. albicans*. Namely, nowadays invasive *Candida* infections are one of the leading causes of mortality in hospitalized and immunocompromised patients. In the present study in vitro techniques have been utilized with the aim to evaluate berberine as a potential antifungal therapeutic and its effects on the membrane and cell wall.

## Methods

### Microorganism

*Candida albicans* strain ATCC 90028 from stock culture collection of the Department of Microbiology, Faculty of Pharmacy and Biochemistry, University of Zagreb was used for all assays performed.

### Berberine preparation

Berberine (chloride form purchased from Sigma, USA) was dissolved in 50% (*v*/v) ethanol to prepare stock solution (5 μg/mL).

### Berberine uptake into *C. albicans* cells

**Fig. 1:**
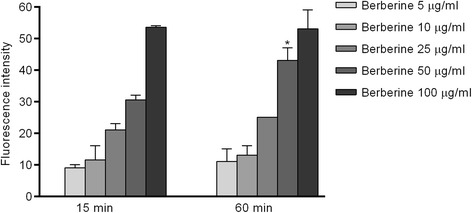
Fluorescence emitted by berberine sulfate upon intracellular localization in *C. albicans* cells. The samples were treated with different berberine concentrations for 15 and 60 min. The data are shown as means ± SD

Intracellular berberine concentration was detected in exponentially growing *C. albicans* cells [9]. Briefly, cells were harvested, washed twice with PBS (Phosphate-buffered saline), and re-suspended at 5 × 10^7^ cells/mL. Different concentrations of berberine (5, 10, 25, 50 and 100 μg/mL) were added. PBS was added to the control tube (not presented in Fig. 1 since this fluorescence was set as background). One milliliter of each sample was incubated at 37 °C for 15 and 60 min, centrifuged, washed twice with PBS, and re-suspended. OD_600_ of each sample was adjusted to 0.1 prior to readings. Fluorescence was read in triplicate in 100 μL of each sample from a black 96-well microplate (Greiner, Germany) with a 405 nm excitation and 520 nm emission (Infinite 200 microplate reader, Tecan Group Ltd., Switzerland).

### Determination of antifungal susceptibility

The minimum inhibitory concentration of berberine as the lowest concentration giving rise to an inhibition of growth of ≥50% of that of the drug-free control against *C. albicans* was assessed according to the method reported by Wei and colleagues [7] with minor modifications. Suspension of *C. albicans* cells was added in sterile flat-bottom 96 well microtiter plate. Serial broth microdilutions of berberine ranging from 256 to 2 μg/mL were added to fungal cells. Plates were incubated aerobically in dark (24 h, 37 °C). Control wells contained 100 μL of cell suspension and berberine solvent. Following incubation XTT (2,3-bis(2-methoxy-4-nitro-5-sulfophenyl)-2H–tetrazolium-5-carboxanilide)/menadione solution (0.5 mg/mL XTT and 1 μM menadione (in acetone)) was added for determination of cell viability. Background absorbance was set as dilution of berberine in RPMI 1640 with 2% glucose with addition of XTT/menadione solution. After incubation (2 h, 37 °C) the absorbance was read at 490 nm (iEMS Reader, Labsystem, Finland) and viability of cells was calculated using equitation:% viability = [A_490_ (treated) - A_490_ (background)]/A_490_ (control) × 100.

The test was performed 5 times and results are presented as mean ± SD (*N* = 5). MIC was calculated using non-linear regression.

### Identification of apoptotic and necrotic cells

Viability of *C. albicans* cells was determined using fluorescent dye exclusion method. The method enables differentiation between viable (intact plasma membrane) and dead cells (damaged plasma membrane) after staining with fluorescent DNA– binding dyes [11]. The assay measures alterations in permeability of individual cell membrane since viable cells exclude ethidium bromide and the appearance of their intact nuclei is bright green. Thus, chromatin in non-viable cells is orange to red colored with organized structure while apoptotic cells are bright green with highly condensed or fragmented nuclei.

In test tubes 100 μL of inoculum suspension (1.5 McFarland units) was mixed with 900 μl of RPMI 1640 with 2% of glucose and different concentrations of berberine (2xMIC, MIC and 1/2xMIC). Amphotericin (1 μg/mL) treated cells served as positive control. The samples were incubated at 35 °C for 3 h. DNA– binding dyes (ethidium bromide and acridine orange) were added to the samples at a final concentration of 100 μg/mL (1:1; *v*/v). Samples were analysed under fluorescent microscope.

### Inhibition of germ-tube formation

The test organism *C. albicans* was cultured on Sabouraud 2% (*w*/*v*) glucose agar (Merck, Germany) for 24 h at 37 °C, aerobically. Inoculum suspension (0.5 McFarland units, nephelometer, bioMerioux, France) for the assay was prepared from fresh culture in physiological saline. The analysis was performed according to the method of Zuzarte et al. [12] with slight modifications. Briefly, test tubes contained 100 μL of inoculum suspension and 900 μL of N-acetyl-D-glucosamine (NAcDG), Leeʾs medium, Spiders medium or yeast-potato-dextrose broth (YPD) + 10% (*v*/v) foetal bovine serum (FBS) with 17.75, 8.75or 4.375 μg/mL berberine. Negative control contained no cells. The samples were incubated at 35 °C for 5 h. Number of yeast cells with germ-tubes, versus non-germinated cells were determined in Neubauer chamber using phase-contrast microscopy.

### Modulation of membrane ergosterol content

The inhibition of ergosterol synthesis was determined in inoculums prepared from fresh cultures of *C. albicans* with different concentrations of berberine (2xMIC, MIC and 1/2xMIC) according to the method of Kumar and Shukla [13]. Sample treated with voriconazole (4 μg/mL) served as positive control. The samples were incubated at 37 °C for 18 h on orbital shaker (170 rpm) aerobically. Following incubation the cells were harvested by centrifugation (2700×g, 5 min) and the weight of the cell pellet was determined. Freshly prepared alcoholic potassium hydroxide solution (25% m/v, 3 mL) was added to each pellet and vortexed vigorously for 1 min. Obtained cell suspensions were transferred to borosilicate glass tubes and incubated for one hour at 85 °C in a water bath and then allowed to cool. The sterol extraction was enabled by addition of water: n-heptane mixture (1:3 *v*/v) followed by vortexing (3 min). The produced heptane layer was transferred to a new borosilicate glass tube with screw-cap. Prior to acquisition, 0.6 mL of sterol extract was diluted in 100% ethanol (1:5) and then scanned between 240 and 300 nm at 5 nm intervals (Varian Cary 1 UV-VIS spectrophotometer, Agilent, USA). Characteristic four-peaked curve is indicative for the presence of ergosterol and the late sterol intermediate 24(28) dehydroergosterol (DHE), while the absence of detectable ergosterol in extracts is presented by a flat line. In addition, dose-dependent decrease in the height of the absorbance peaks may be seen and corresponds to a decrease in ergosterol concentration. In our experiments we calculated the ergosterol content as a percentage of the wet weight of the cell using equations:

$$ \begin{array}{c}\hfill \%\mathrm{ergosterol}+\%24(28)\ \mathrm{DHE}=\left[\left({\mathrm{A}}_{281,5}/290\right)\ \mathrm{x}\ \mathrm{F}\right]/\mathrm{cell}\ \mathrm{mass}\hfill \\ {}\hfill \%24(28)\ \mathrm{DHE}=\left[\left({\mathrm{A}}_{230}/518\right)\ \mathrm{x}\ \mathrm{F}\right]/\mathrm{cell}\ \mathrm{mass}\hfill \\ {}\hfill \%\mathrm{ergosterol}=\left[\%\mathrm{ergosterol}+\%24(28)\ \mathrm{DHE}\right]\hbox{-} \%24(28)\ \mathrm{DHE},\hfill \end{array} $$

where F is the factor of sample dilution in ethanol (1:5) and 290 and 518 are the E values (in percentages per centimeter) determined for crystalline ergosterol and 24 (28) DHE, respectively.

### Modulation of cell membrane permeability

The effect of berberine on *C. albicans* cells was further evaluated at the level of cell membrane integrity using the method of Khan and coworkers [14]. To analyze the possible effect of berberine on the cell wall, we tested the releasing of the crucial cell content using spectrophotometric measurement of cell supernatant at 260/280 nm (corresponding to nucleic acids and proteins). Cell suspensions prepared from fresh cultures of *C. albicans* (2.5 × 10^7^ CFU/mL) were treated with different concentrations of berberine (10xMIC, 2xMIC and MIC) for different time intervals (1 h, 3 h, 6 h, 12 h, and 24 h). Positive control was performed with voriconazole (4 μg/mL). After incubation period, the samples were centrifuged (1250 rpm, 2 min) and the release of cellular material in the supernatants was determined (Biospec Nano, Shimadzu, USA).

### Depolarisation of plasma membrane

The effect of berberine treatment on vitality of *C. albicans* cells was investigated using bis-(1,3-dibutylbarbituric acid) trimethine oxonol [DiBAC_4_(3)] (Molecular Probes, USA) dye [15]. This method allows monitoring of possible changes in the polarisation state of the cell membrane.

The dye DiBAC_4_(3) (final concentration 2 μg/mL) was added to the aliquots of cell suspensions (10^7^ CFU/mL) pretreated (60 min) with different concentrations of berberine (1/2xMIC, MIC and 10xMIC).The incubation with the dye lasted 1 h in the dark following washing with PBS. Fluorescence intensity was measured with 488 nm excitation and 510 nm emission (Infinite 200 microplate reader, Tecan Group Ltd., Switzerland).

### Determination of lipid peroxidation in whole cell

To determine the level of lipid peroxidation, malondialdehyde (MDA) level was measured by reaction with thiobarbituric acid reactive substances (TBARS) [15]. After treatment with berberine (1/4xMIC, 1/2 × MIC, MIC) for 4 h *C. albicans* cell suspension was centrifuged (12,000×g, 5 min), the pellet was re-suspended in lyses buffer (2% Triton-X 100, 1% SDS, 100 mM NaCl, 10 mM Tris–HCl, 1 mM EDTA [pH 8.0]) and sonicated on ice. Following centrifugation (12,000×g, 2 min) thiobarbituric acid (TBA, 0.5% *w*/*v*) solution in trichloroacetic acid (TCA, 5%) was added (1:1) to the supernatant. The mixture was heated (95 °C, 60 min) and then cooled on ice. Following centrifugation (10,000×g, 10 min, 4 °C) the absorbance of the supernatant fraction was determined at a wavelength of 532 nm and 600 nm. All experiments were done in triplicate. The protein level was determined using BSA as standard [16].

### Determination of lipid peroxidation in plasma membrane preparation

To determine the level of lipid peroxidation of plasma membranes of *C. albicans* cells malondialdehide (MDA) level was measured as described above [15].

Cells were grown in YPD broth containing different concentrations of berberine (1/4 × MIC, 1/2 × MIC, MIC) at 30 °C with shaking. Following cell disruption (homogenizing buffer: 2 mM EDTA, 20% glycerol (*v*/v), 1 mM phenylmethylsulphonyl fluoride (PMSF) and 50 mM Tris, pH 7.5), homogenate was centrifuged twice (2000×g, 10 min) with washing between. The pelet was resuspended and the plasma membrane fractions were obtained by centrifugation (55,000×g, 45 min). The pellet containing plasma membranes was suspended (20% glycerol *v*/v, 0.5 mM EDTA, 0.5 mM PMSF, 10 mM HEPES, pH 7.0) and washed once by centrifugation and stored until assay. The protein level was determined using BSA as standard [16].

### Measurement of ROS levels

The effect of berberine (1/2xMIC and 1 × MIC) treatment on intracellular ROS levels in *C. albicans* cells was assessed using the fluorescent dye chloromethyl-dichlorodihydrofluorescein diacetate (CM-H_2_DCFDA) [17]. Briefly, after treatment with different concentrations of berberine CM-H_2_DCFDA (final concentration: 20 μM) was added to the cells and incubated (37 °C, 1 h). Fluorescence intensity was measured with a 485 nm excitation and 535 nm emission (Infinite 200 microplate reader, Tecan Group Ltd., Switzerland).

### Statistical analysis

The experiments were performed as triplicates at least three times at independent occasions. Results are presented as the mean ± standard deviation where appropriate. Statistical analyses were performed using GraphPad Prism 4.0 software and *p* < 0.05 was considered statistically significant.

## Results

### Berberine uptake into *C. albicans* cells

To test the possible accumulation of berberine inside the *C. albicans* cells we exposed them to berberine at different concentrations and two time points. The results (Fig. 1) showed an almost linear increment of fluorescence intensity along with dose increase (dose-dependent berberine accumulation). It is also observed that berberine accumulation at a dose 50 μg/mL is time-dependent (augmentation of fluorescence intensity following 60 min- versus 15 min-treatment).

### Determination of antifungal susceptibility

**Fig. 2:**
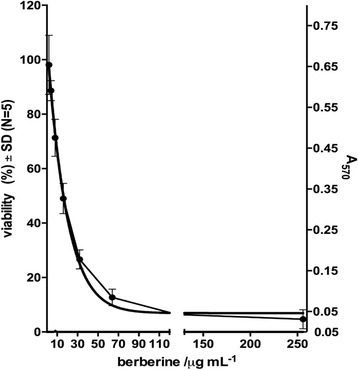
Non-linear regression line assessing viability of *C. albicans* treated with berberine. The data are shown as means ± SD

Using tetrazolium salt (XTT) reduction assay, the viability of *C. albicans* cells treated with serial, two-fold dilution of berberine is shown in Fig. 2. Using non-linear regression, drop of viability up to 50% in comparison to the control (untreated) cells was estimated as MIC. Determined MIC value was 17.75 μg/mL.

### Identification of apoptotic and necrotic cells

Quantitative fluorescent-dye exclusion test was used to assess cell death of *C. albicans* treated with berberine in vitro for 3 h. Results of the assay show that berberine significantly (*p* < 0.05, Pearson chi-square test) reduced cell viability compared to the negative control at all concentrations used (1/2 × MIC; MIC; 2xMIC). (Table 1) The observed effect was concentration dependent (Pearson chi-square test *p* < 0.05).

**Table 1:** Results of the quantitative fluorescent assay for simultaneous identification of apoptotic and necrotic cells in *C. albicans* ATCC 90028 treated with berberine in vitro for 3 h

Sample	Viable cells (%)	Non-viable cells		
		Σ	Apoptosis (%)	Necrosis (%)
½ MIC	88.7 ± 2.1	11.3 ± 2.1^MIC,2xMIC,NC,PC^	7.7 ± 1.5 ^MIC,2xMIC,PC^	3.7 ± 2.5^2xMIC,PC^
MIC	75.3 ± 2.5	24.7 ± 2.5^2xMIC,NC,PC^	18.0 ± 1.7^2xMIC,NC,PC^	6.7 ± 1.2^NC^
2× MIC	62.3 ± 9.7	37.7 ± 9.7^NC^	28.3 ± 10.3^NC^	9.3 ± 0.6^NC^
PC	56.0 ± 4.6	44.0 ± 4.6^NC^	35.3 ± 2.3^NC^	8.7 ± 2.5^NC^
NC	94.7 ± 1.2	5.3 ± 1.2	4.0 ± 1.7	1.3 ± 0.6

300 cells per sample per each experimental point were analysed. Mean values ± SD are shown. *MIC* minimal inhibitory concentration, *PC* positive control, *NC* negative control (RPMI). Statistical significance of data was evaluated using χ^2^ test. The level of statistical significance was set at *P* < 0.05. The abbreviations next to the means indicate from which groups the relevant group differs with statistical significance

### Inhibition of germ-tube formation

**Fig. 3:**
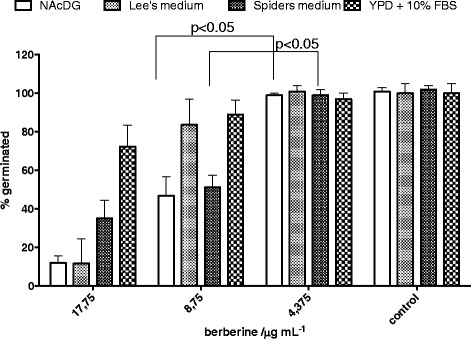
Effect of different concentrations of berberine on germ tube formation in *C. albicans*; NC – intact cells. The data are shown as means ± SD

After incubation at 35 °C for 5 h statistically significant (*p* < 0.05) inhibition of morphological transition of *C. albicans* cells to its filamentous form was observed for samples treated with two concentrations (1/2 × MIC and1/4 × MIC) of berberine in comparison to the negative control. According to data shown in Fig. 3, the inhibitory effect of berberine at concentration 8.75 μg/mL (¼xMIC) was noticed in media containing NAcDG and Spider’s medium. On the other hand, in Lee’s media and in YPD media with addition of 10% of fetal bovine serum the effect of berberine was less pronounced. The results suggest that berberine affects two different metabolic pathways which regulate budded-to-hypha transition in vitro.

### Modulation of membrane ergosterol content

**Fig. 4:**
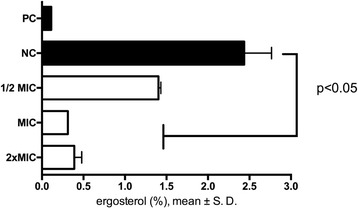
Modulation of ergosterol content at different concentration of berberine; PC-voriconazole 4 μg/mL^1^; NC-intact cells. The data are shown as means ± SD

The effect of berberine on the membrane of *C. albicans* cells was assessed using ergosterol synthesis assay. Figure 4 shows modulation of ergosterol biosynthesis at 1/2 × MIC, MIC and 2xMIC concentration of berberine. Berberine modulates ergosterol content significantly (*p* < 0.05) in a concentration dependent manner. At the lowest concentration (1/2 × MIC) berberine caused 39% reduction in total sterol content, while two other concentrations produced a reduction of 84 and 87%, respectively.

### Modulation of cell membrane permeability

**Fig. 5:**
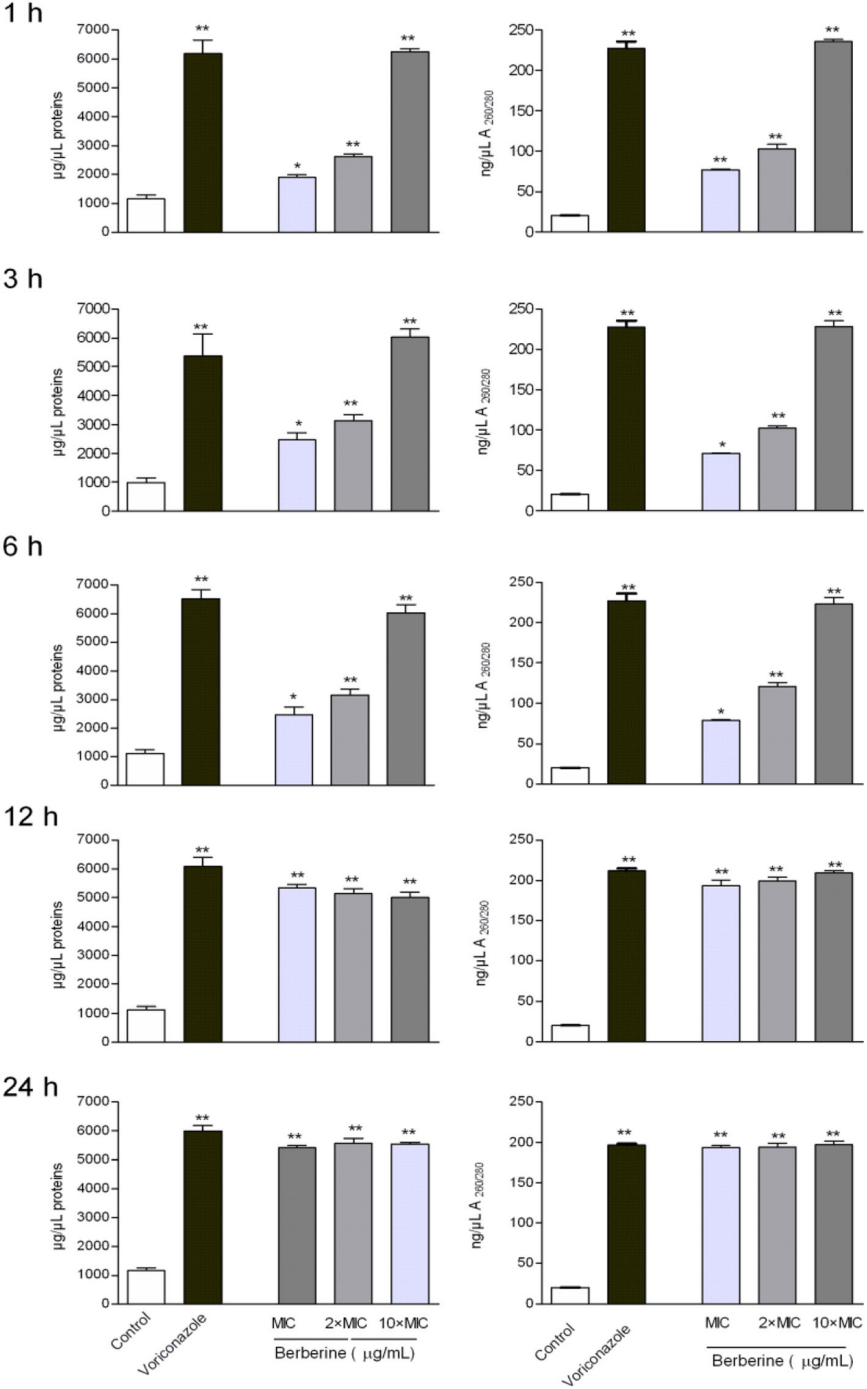
The effect of berberine on cell permeability and integrity of cell membranes of *C. albicans* cells. The data are shown as means ± SD

We measured the effect of berberine on cell permeability and integrity of cell membranes. Spectrophotometric measurements of intracellular components that absorb at 260 nm (nucleotides) and 280 nm (protein) in the cell supernatant revealed time- and dose-dependent effect of berberine on the cell membrane permeability. The results show release of intracellular components to the extracellular compartment (Fig. 5). As shown in Fig. 5 berberine, at all concentrations tested, significantly damaged the fungal cell wall within 60 min of treatment causing subsequent increase in DNA/RNA and protein content in extracellular media. Similar trend was observed at other time points with two lower berberine concentrations while the highest berberine concentration (10 × MIC) caused membrane damage within the first hour of treatment. This effect was similar to the effect of voriconazole, which served as positive control. It seems that berberine at highest concentration used, similar to voriconasole, produced maximal possible damage. On the other hand, two lower berberine concentrations reached its plateau of action following 12 h treatment period (there is no further increase in the cellular content outside the cells in following time points assessed and therefore only the last point -24 h - is shown on the graph).

### Depolarisation of plasma membrane

**Fig. 6:**
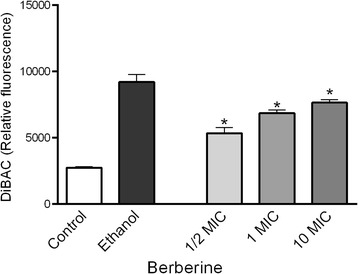
The effect of different berberine concentrations on membrane depolarisation of *C. albicans* cells as a measure of membrane damage and cell viability. The data are shown as means ± SDs

We used DiBAC_4_(3), dye which permeates depolarized cell membranes and binds to intracellular proteins with consequent fluorescence enhancement, to further asses the effect of berberine on the membrane of *C. albicans* cells. Staining of *C. albicans* with this dye revealed a significant (*p* < 0.05) increase in the relative fluorescent units in cell suspensions incubated for 1 h with berberine at all three concentrations (Fig. 6). The effect was dose-dependent (slight increase of relative fluorescent units with a dose increase) although there was no significant change between different concentrations used.

### Lipid peroxidation levels

Upon the treatment of *C. albicans* cells with berberine (three different concentrations) the results showed enhancement of lipid peroxidation levels. Moreover, we isolated the membranes of those cells and the peroxidation level of membrane lipids of *C. albicans* cells showed that berberine up-regulates significantly (*p* < 0.05) MDA levels in all treated groups (1/4 × MIC, 1/2 × MIC, and MIC).

### Level of ROS following berberine treatment in *C. albicans*

**Fig. 7:**
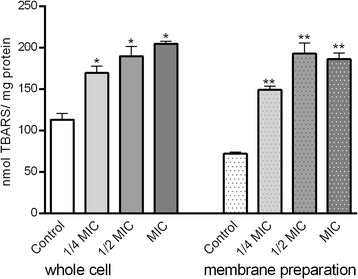
Lipid peroxidation levels in control and berberine-treated cells of C. albicans and their isolated membranes. Berberine induced dose-dependent increase in TBARS /MDA level as a measure of lipid peroxidation. Cells were treated with berberine (1/4×MIC, ½×MIC and 1×MIC) and the level of lipid peroxidation was assessed in complete cells and membrane preparations. MDA concentration was determined using the TBARS assay. The data are presented as mean values ± SD (*p < 0.01; **p < 0.05; ***p < 0.001).

Changes in ROS generation upon berberine treatment of *C. albicans* cells was assessed using fluorescent molecule CM-H_2_DCFDA which is sensitive to redox changes. The dye enters the cells and upon deacetylation into dichlorofluorescein emits fluorescence upon oxidation by ROS. Berberine enhanced fluorescence (which reached statistical significance following the treatment with MIC concentration) indicating generation of ROS in treated cells (Fig. 7).

## Discussion

There is a large gap between needs and available treatments especially in terms of antimicriobial drugs and there are significant efforts to fill this gap with substances of natural origin [1, 2]. One of the hard-to-treat infections is candidiasis and, based on the previous studies [9, 10], our search has been directed to the effectiveness and explanation of mechanisms of action of alkaloid berberine. We show that berberine may enter *C. albicans* cell (Fig. 1) and may act not only from extracellular site but also inside the fungus cell, having significant antifungal activity against *C. albicans* with MIC value of 17.75 μg/mL.

*C. albicans* is a polymorphic fungus and is able to covert to the filamentous form what represents a virulence mechanism which plays an important function in host tissue invasion and resistance to phagocytosis [18]. It has been reported that fungal invasion is facilitated more by the transition between yeast cells and filamentous growth than by yeast growth itself [19]. Morphogenetic transition is a phenomenon which occurs in response to external stimuli including elevated temperature or pH, nitrogen and/or carbon starvation, and the presence of the host macrophages [20]. We tested the effect of berberine against *C. albicans* in variety of hyphal-inducing media and observed inhibition of filamentation in all media used. However berberine was less effective in YPD media supplemented with 10% FBS suggesting that serum constituents may affect tested compound or interfere with its action. Most prominent berberine effect was observed at subMIC concentration when culturing in NAcDG containing media in which MAPK pathway of morphogenesis is triggered. Berberine also inhibited germ-tube formation of *C. albicans* cells in sub-MIC concentration in Spider’s medium where the transition is mediated by cAMP-PKA pathway. Namely, inhibition of germ-tube formation of *C. albicans* by berberine was stronger at sub-MIC concentration in media where MAPK and cAMP-PKA pathways of budded-to-hypha transition are employed. On the other hand, in Lee’s media (Cph2 to Tec1 regulation of hyphal transition) and in yeast-potato-dextrose media with addition of 10% of fetal bovine serum the effect of berberine was less pronounced. This implies the interference of berberine in metabolic pathways what needs further clarification [20].

Anti-*Candida* activity was assessed with fluorescent-dye exclusion test which enables differentiation between viable blastospores, which exclude ethidium bromide having bright green nuclei with an intact structure, and non-viable cells which have orange to red chromatin with organized structure. Apoptotic cells are bright green with highly condensed or fragmented nuclei [21]. Penetration of ethidium bromide into the cell indicates disruption of the membrane integrity as a possible mechanism of berberine action. Berberine also induced time- and dose- dependent leakage of DNA and proteins from inner to the extracellular space (Fig. 5). Namely, leakage of low molecular weight cytoplasmic components may be an indicator of the membrane disorganization [22]. Similar was noticed for *S. agalactiae* where berberine induced serious damage of cell membrane and cell wall, and consequently resulted in the reduction of protein materials within the cells [23]. We used anionic lipophilic dye DiBAC_4_(3) to assess the effect of berberine on the membrane potential as cells at physiological state exclude the dye (negative internal charge) and damaged cells have depolarized membrane and the dye enters the cell, binds to the lipid-rich intracellular components causing increase in the fluorescence. Our experiments showed dose-dependent increase in relative fluorescence units (Fig. 6) in the berberine-treated cells and strengthen our hypothesis on the possible effect of berberine on the activity at cell membrane subsequently leading to the cell death, probably due to apoptosis [17, 24] as indicated in a study on fluconazole-resistant strains [10]. Data presented indicate that berberine may cause apoptosis in *C. albicans* cells as studies suggest that ROS accumulation induces and/or regulates the induction of apoptosis in yeasts [17, 25].

Ergosterol maintains membrane fluidity and is involved in membrane lipid arrangement. Decrease in its content following berberine treatment may lead to the loss of membrane permeability and thus induce cell vulnerability or even cell death [26, 27].

**Fig. 8:**
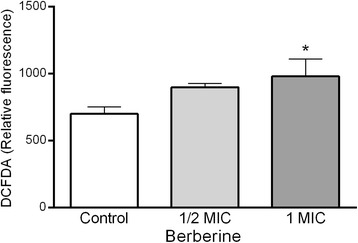
Changes in ROS generation upon berberine treatment of C. albicans cells. Berberine at sub MIC concentration induced ROS production. The augmentation of ROS production reached statistical significance upon the treatment with MIC concentration of berberine. The data are presented as mean values ± SD (*p < 0.05).

Using TBARS assay we showed the accumulation of reactive species including hydroxyperoxides and aldehydes, which are indicators of lipid damage [28]. The significant increase of TBARS in berberine treated cells, and specifically in their membrane preparation (Fig. 8), is a sign of an oxidative stress. These results are in line with the analyses of ethidium bromide incorporation into the cells (Table 1). Taken this together with the finding that berberine inhibits ergosterol synthesis (Fig. 4), berberine may have dual effect on the lipid peroxidation of the membrane content. Namely, ergosterol is needed not only for maintenance and regulation of the structural and functional integrity of the fungal membrane but also inhibits lipid peroxidation [29]. Thus, since berberine inhibits ergosterol and induces oxidative stress, it may have aggregated effect on lipid peroxidation levels in *Candida* cells. This mechanism of berberine action may also explain the permeabilisation of the membrane and the incorporation of ethidium bromide. To further characterize the effect of berberine regarding these events in *Candida* cells it would be needed to assess activity levels of SOD and catalase as a response and a defense mechanism at enhanced ROS levels [14, 17, 27, 30]. Namely, all organisms/cells are permanently affected by reactive oxygen and nitrogen species but oxidative stress and its consequences occur only when the cell is not able to overcome its „overload“[31]. *C albicans* plasma membrane is composed of app 70% polyunsaturated lipids [32]. High level of lipid peroxidation products following different noxious is predictable [11, 33]. Moreover, lipid peroxidation may lead to the functional and functional changes of the plasma membrane, and at higher extent, to the cell death [24, 27, 34]. In addition, Dhamgaye and colleagues [9] showed that berberine treatment results in dysfunctional mitochondria, which was evident from its slow growth in non-fermentative carbon source. They also showed poor labeling of treated cells with mitochondrial membrane potential sensitive probe [9] confirming further possible use of berberine as antifungal drug.

Our findings suggest that berberine may change sterol profile of yeast by causing inhibition of ergosterol biosynthesis. Berberine also induces lipid peroxidation which may be one of the mechanisms involved in its *Candida*-cidal activity.

## Conclusions

Based on the results presented, we conclude that berberine induces mechanisms involved in its *Candida*-cidal activity probably mainly at the level of the cell membrane. Therefore it seems that berberine may serve as an alternative for the treatment and/or prevention of candidiasis.

## Abbreviations

ATCC: American type culture collection
BCA: Bicinchoninic acid
CFU: Colony*-*forming unit
DCFDA: 2′,7′-Dichlorodihydrofluorescein diacetate
DHE: Dehydroergosterol
DiBAC4: Bis-(1,3-Dibarbituric acid)-trimethine oxanol
DNA: Deoxyribonucleic acid
EDTA: Ethylenediaminetetraacetic acid
EUCAST: European committee on antimicrobial susceptibility testing
FBS: Fetal bovine serum
HEPES: (4-(2-hydroxyethyl)-1-piperazineethanesulfonic acid
MDA: malondialdehyde
MDR: Multidrug-resistant
MIC: Minimum inhibitory concentration
MRSA: Methicillin-resistant *Staphylococcus aureus*
NacDG: N-Acetyl-D-Glucosamine
PBS: Phosphate-buffered saline
PMSF: phenylmethylsulphonyl fluoride
ROS: Reactive oxygen species
RPMI: Roswell park memorial institute
SOD: Superoxide dismutase
TBARS: Thiobarbituric acid reactive species
TCA: Trichloroacetic acid
XTT: 2,3-Bis-(2-methoxy-4-nitro-5-sulfophenyl)-2H–*tetrazolium*-5-carboxanilide salt
YPD: Yeast potato dextrose
